# An IoT BLE based system literature review of real time location monitoring and tracking to shorten patient wait times in Malaysian public hospitals

**DOI:** 10.12688/f1000research.160992.1

**Published:** 2025-06-09

**Authors:** Ganes Raj Muthu Arumugam, Kalaiarasi Sonai Muthu Anbananthen, Saravanan Muthaiyah

**Affiliations:** 1 Faculty of Management, Multimedia University, Persiaran Multimedia,, Cyberjaya, Selangor, 63100, Malaysia; 2 Faculty of Information Science and Technology, Multimedia University, Jalan Ayer Keroh Lama, Bukit Beruang, Melaka, 75450, Malaysia; 3 School of Business and Technology, International Medical University, 126, Jln Jalil Perkasa 19, Bukit Jalil, Kuala Lumpur, 57000, Malaysia

**Keywords:** Patients Turnaround Time (PTAT), BLE, IoT, Public Hospital; Real Time Monitoring

## Abstract

One important performance metric in healthcare, particularly in Malaysia’s public hospitals, is patient turnaround time (PTAT). It describes the time that passes between receiving a patient’s request or sample and making a diagnosis or treatment decision. To guarantee prompt patient care, efficient treatment planning, and general healthcare quality, PTAT optimization is essential. Delays in public hospitals are often caused by complex procedures and inefficiencies in patient flow, particularly the lack of tracking when patients leave waiting areas. This study conducts a systematic literature review (SLR) using the PRISMA methodology to examine existing research on PTAT in healthcare. The findings highlight critical factors affecting PTAT in Malaysian hospitals, emphasizing the need for improved patient tracking systems to enhance operational efficiency and healthcare service delivery. It pinpoints recurring themes, knowledge gaps, and possible improvement opportunities. By knowing the elements driving PTAT, administrators, policymakers, and healthcare professionals can be better equipped to adopt measures for improvement. To overcome PTAT difficulties, our study emphasizes the significance of a real-time patient localization system utilizing Bluetooth Low Energy (BLE). This internet of things (IoT) based solution provides a unified strategy for enhancing turnaround times by enabling the real-time tracking of patient movements. The proposed method aims to achieve a PTAT of less than an hour by reducing waiting times by 35% through the integration of current technologies into a unified system. Achieving this objective would enhance the overall effectiveness of patient treatment and drastically reduce missed visits.

## 1. Introduction

The swift rate of urban growth and technological progress has compelled numerous people to move to cities, resulting in heightened congestion. ^1^ As individuals pursue better living standards, especially in healthcare, these metropolitan areas have become more crowded. In Malaysia, as of 2019, approximately 74.84 percent ^2, 3^ of the population lived in urban regions, a number that has been steadily increasing. This demographic change has led to numerous challenges in the healthcare system, such as hospital overcrowding and a lack of medical supplies. ^1^ These circumstances worsen wait times for patients who frequently experience an extended period of uncertainty while waiting for medical care. While waiting, patients might unintentionally stroll around the hospital grounds, making it more challenging for staff to find them. This issue worsens when patients are summoned by doctors from various departments, resulting in confusion and aimlessness throughout the facility. The effects of this inefficiency exceed those of single patients, resulting in longer wait times for others, ultimately reducing overall productivity in the healthcare system. The difficulties associated with patient navigation in hospitals emphasize the necessity for creative solutions, such as the adoption of real-time Internet of Things (IoT) technologies that leverage Bluetooth Low Energy (BLE). In Malaysia’s public hospitals, these technologies can enhance patient tracking and management, streamline procedures, and speed up patient turnaround times. Healthcare facilities can improve service quality and reduce overcrowding issues by adopting these technological advancements, which would benefit both patients and medical staff.

Optimizing hospitals is crucial for enhancing patient care through effective resource use and boosting overall productivity. ^4^ A major challenge encountered by healthcare facilities today is the struggle for staff to track or locate patients across hospital grounds. In numerous instances, despite a patient being in the facility, they might miss their appointment because of communication issues or staff not finding them promptly. Patients, physicians, and the general public become dissatisfied as a result of such inefficiencies, which aggravate both patients and healthcare professionals. Ineffective management of time, staff, and medical equipment results in preventable delays and reduces a hospital’s ability to provide more patients with timely care. To address these issues, it is essential to adopt strategies that enhance both care quality and resource management. Hospitals need to adopt cutting-edge technologies and contemporary solutions to improve their operational efficiency, reduce patient wait times, and guarantee streamlined workflows among departments. As the demand for healthcare services increases, improving hospital operations has become essential to fulfilling public expectations and sustaining service quality.

Although there have been major advancements in healthcare technology, scant research and solutions have effectively merged localization and monitoring into one complete system to enhance the Patient Turnaround Time (PTAT). Attaining this integration is complicated because it encompasses numerous technical and operational factors that need to function smoothly in a hospital environment. ^5^ Monitoring requires wearable devices with internet connectivity to supply real-time patient location information. This infrastructure includes additional crucial elements such as sensors, storage solutions, and communication networks. Conversely, monitoring extends past basic tracking and includes tasks such as data gathering, analysis, reporting, and ongoing observation to extract actionable insights from patient movement trends and hospital functions. Due to the variety and intricacy of hospital settings, which vary in organization, patient movements, and departmental functions, creating a cohesive solution that accommodates these factors is especially difficult. This complexity underscores the difficulty in achieving a completely integrated system for PTAT reduction. Nonetheless, as the globe moves towards an era of Smart Healthcare, the demand for such a solution has grown more pressing to enable effective, high-quality healthcare provision.

**Figure 1.**
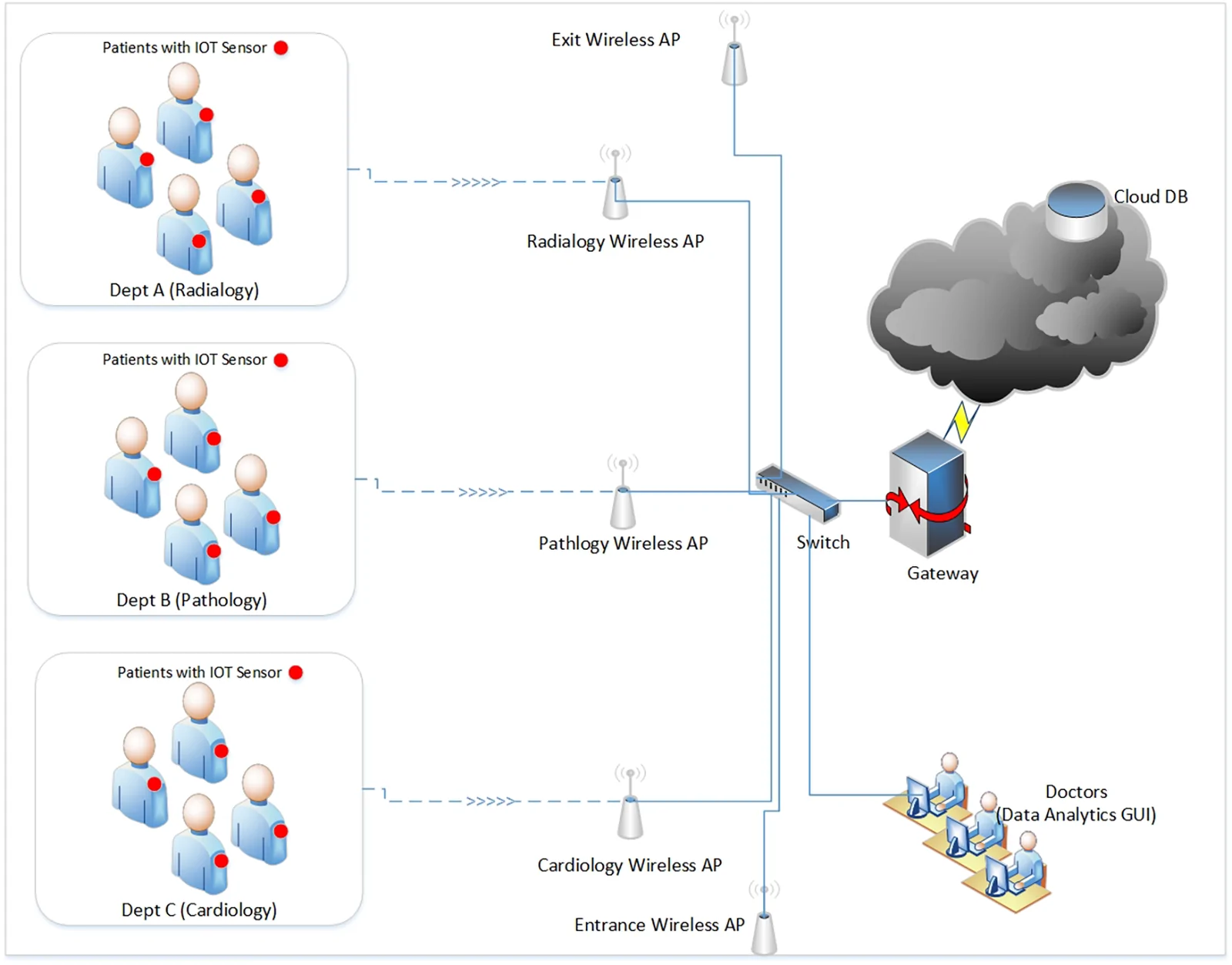
Flow of real time patient location monitoring and tracking solution.

A comprehensive and efficient real-time patient location tracking system can be developed utilizing two primary technologies: Bluetooth Low Energy (BLE) and the Internet of Things (IoT). ^6^ Both technologies perform unique, but interconnected roles. BLE enables low-energy and ongoing monitoring of patient movements across hospital grounds. The IoT facilitates the gathering, storage, and immediate analysis of data to oversee patient movement and enhance hospital efficiency. In the suggested approach, when patients arrive at the hospital, they receive a BLE-enabled bracelet that holds critical information including the patient’s ID, name, and additional pertinent details. As the patient navigates various sections of the hospital, strategically located readers or Access Points (APs) mounted on walls or ceilings pick up signals from BLE tags. The gathered information is sent to a cloud database, where it is instantly analyzed and processed. This system enables staff to monitor patient flow between departments, guaranteeing that they are effectively guided to their appointments. It additionally aids staff in quickly addressing situations when a patient does not show up for an appointment or is located in an incorrect department, thereby reducing delays and enhancing resource management. Figure 1 shows the tracking flow.

Decreasing Patient Turnaround Time (PTAT) greatly improves the total efficiency and quality of healthcare services by facilitating smooth transitions between departments with little to no delay. Real-time tracking systems enhance staff efficiency by enabling hospital employees to concentrate on patient care instead of searching for individuals, whereas insights based on data streamline resource management by recognizing trends in patient movement and departmental needs. This leads to improved distribution of personnel, resources, and facilities, ultimately fostering a more seamless operational process. Additionally, prompt care and effective navigation throughout the hospital significantly boost patient satisfaction, as reduced wait times and smoother processes elevate the overall experience. This solution tackles the logistical issues of overseeing patient transfers, establishing a basis for an interconnected healthcare ecosystem driven by IoT and Bluetooth low-energy (BLE) technology. As hospitals implement these advanced systems, they open new avenues for attaining operational excellence, providing exceptional service quality, and maintaining a patient-focused approach to care, thus influencing the future of innovative and sustainable healthcare.

### 1.1 Defining real time patience location monitoring and tracking solution

Real-time location systems (RTLS) are designed to dynamically detect and map an individual’s location within enclosed spaces such as hospitals or other healthcare facilities in real time. These systems typically rely on wireless wearable tags attached to individuals or objects that emit signals picked up by fixed reference access points throughout the building. The collected data helps determine precise locations, enabling seamless tracking and monitoring of people and assets within the facility. Although Global Positioning Systems (GPS) are effective for outdoor real-time tracking, their accuracy and reliability degrade significantly indoors because of signal obstructions. ^7^ To overcome these challenges, healthcare providers use a range of location awareness technologies to support indoor monitoring and ensure smooth operation within medical environments. The ability to monitor the movement of patients, staff, and equipment has numerous applications in healthcare, contributing to improved workflow, reduced wait times, and enhanced patient care. This study provides a simplified primer for real-time patient location monitoring and tracking solutions in healthcare settings. It discusses the variety of technologies and innovations involved, their practical applications, and their future benefits. These advantages include maintenance cost savings, improved operational workflow, and higher patient satisfaction. However, the success of an RTLS solution depends on the choice of the right technology for a specific application or problem.

The cornerstone of effective RTLS implementation is to align the appropriate technology with the desired application. For instance, BLE is well-suited for real-time patient monitoring, whereas UWB may be better for tracking essential equipment that requires accurate location information. ^8^ Not aligning the technology with a particular operational requirement may lead to less-than-ideal results or the potential failure of the system. With healthcare facilities increasingly adopting digital transformation and intelligent healthcare technologies, RTLS will be crucial for enhancing operations and elevating service delivery. Future innovations in IoT and AI will significantly improve the functions of location-based systems, creating new opportunities for predictive analytics and personalized healthcare. In conclusion, the effectiveness of real-time patient location monitoring and tracking solutions depends on the choice of appropriate technology for a particular application. Careful evaluation of the operational requirements, technological alignment, and future objectives is crucial to guarantee that the solution yields significant outcomes. Through effective planning and implementation, RTLS can significantly improve patient outcomes and hospital efficiency, establishing it as a fundamental aspect of contemporary healthcare.

Real-time patient location monitoring and tracking solutions aim to pinpoint and track the exact whereabouts of individuals, mainly patients, in healthcare settings. These systems employ dedicated fixed receivers or readers (location sensors) that interact wirelessly with badges or tags worn by patients to pinpoint their precise location within the facility. These solutions provide immediate insight into patient movement, aiding workflow optimization, minimizing delays, and enhancing care delivery. Every wearable tag sends a distinct ID to the patient to which it is assigned. As patients progress through different locations such as the imaging department, MRI suite, or consultation rooms, tags consistently transmit their IDs. Receivers or access points positioned strategically across the hospital capture these signals and send the information to the central tracking system. The position of every patient is subsequently established based on the tag’s closeness to the designated sensors, with the system refreshing the patient locations in various zones in real time. Real-time patient location monitoring and tracking solutions are crucial assets for contemporary healthcare, offering insights into patient movements to optimize operations and improve care quality. Although they may not provide ongoing navigation or spatial awareness, these systems are still crucial for resource management, workflow improvement, and patient contentment. Upcoming developments in tracking technology, such as the incorporation of IoT- and AI-driven analytics, could ultimately address existing constraints, ^9^ providing even more accurate and practical insights regarding patient movement and hospital functions.

### 1.2 Real time patience location monitoring and tracking solution components and technologies

The efficiency of a real-time patient location monitoring and tracking system in enhancing Patient Turnaround Time (PTAT) depends on smooth collaboration among its elements and technologies. The system combines different hardware and software components to constantly track patient locations and enhance workflow in healthcare settings. Tags are wearable gadgets (such as badges or wristbands) with unique IDs assigned to patients. Location Sensors are stationary receivers or access points deliberately placed around the facility to capture signals from tags. These sensors served as reference markers for identifying the precise locations of tagged individuals inside the hospital. The location engine serves as the main software tasked with determining the positions of tagged items or individuals. It gathers information from tags and location sensors and analyzes the signal intensity, closeness, or triangulation outcomes to identify the location of each patient. The engine guarantees that real-time tracking information is consistently accessible and refreshed as patients transition between various departments or areas.

The tools used by hospital personnel offer immediate visualization and oversight of patient placement and conditions. These systems enable administrators to monitor patient movements, organize appointments, optimize resource allocation, and address possible bottlenecks in patient flow. ^10^ Applications can consist of mobile platforms, desktop interfaces, or notification systems, allowing the rapid retrieval of location information on various devices. A real-time patient location monitoring and tracking system depends on the collaboration of tags, sensors, location engines, middleware, and applications to enhance the patient turnaround efficiency. The middleware functions as a vital “sensor” between the hardware and software, guaranteeing that precise and usable data can move effectively through the system. When applied successfully, these solutions not only boost operational efficiency, but also elevate patient care and satisfaction, rendering them vital resources for contemporary healthcare institutions.

The variety of solutions for real-time patient location monitoring and tracking includes basic end-user interfaces and sophisticated integrations with large-scale systems, such as public hospital information networks. This variety emphasizes the adaptability and scalability of location-based systems, rendering them appropriate for both personal users and intricate organizational settings. On the one hand, user interfaces offer simple tools for instantly retrieving location information. These interfaces focus on user friendliness, allowing non-technical individuals, such as nurses, administrative personnel, or visitors, to effortlessly search for and access pertinent information without requiring specialized knowledge. These tools improve daily operations by providing a clear and user-friendly interface and reducing delays in finding patients, personnel, or tools. Conversely, these tracking solutions can be incorporated into larger enterprise systems such as hospital information management platforms. These advanced integrations enable location data to be aligned with electronic health records (EHRs), ^11^ patient-scheduling systems, and various business applications, offering an all-encompassing perspective on operations. This enhanced level of integration facilitates improved resource distribution, operational strategies, and instantaneous decision making. For instance, real-time location monitoring can activate automated alerts when patients are late, enabling staff to quickly step in to uphold service quality and reduce waiting periods.

By utilizing these adaptable solutions, healthcare organizations can meet various needs across diverse contexts and stakeholders. Basic, independent interfaces are designed for individual users wanting easy access to location information, whereas integrated solutions address the needs of hospital administrators and IT departments by overseeing intricate workflows. This wide-ranging applicability guarantees that the system remains pertinent across multiple departments, from outpatient services to emergency care, and facilitates long-term scalability as hospital operations expand. Finally, the range of solutions provides both operational ease and technical complexity, allowing hospitals to optimize patient flow, elevate care standards, and boost overall efficiency. Along with simple user interfaces, real-time patient location monitoring and tracking solutions also encompass intricate integrations with organizational networks, including public hospital information systems. These complex interactions facilitate smooth communication and data transfer between the tracking solution and hospital’s current infrastructure. By means of this interoperability, the tracking system can utilize current data sources, workflows, and scheduling systems to further optimize operations, boost coordination among departments, and enhance the overall effectiveness. For instance, the movement of patients can be aligned with scheduling systems, ensuring that staff are notified when patients are on their way or running late, which helps lessen interruptions and reduce waiting periods. The flexibility of these location-based solutions extends beyond user-interface convenience and technical connectivity. They are intended to function in a wide range of settings and user needs, making them relevant not only for healthcare facilities, but also for sectors such as manufacturing, logistics, and retail. This adaptability guarantees that organizations can utilize the technology in various settings, tackling both operational obstacles and specific industry issues. In hospitals, these systems can effectively manage patient movement, staff distribution, and resource use, assisting administrators in proactively addressing shifting circumstances. ^12^ Meanwhile, in different environments such as production facilities, comparable monitoring systems can monitor equipment usage and optimize workflows.

By applying solutions that span user-friendly interfaces to complex system integration, healthcare providers can customize the technology to meet specific challenges. Whether via a straightforward dashboard that assists nurses in finding a patient instantaneously or an intricate integration with electronic health record (EHR) systems for unified management, these tools enable healthcare professionals to make educated choices and provide prompt care. Ultimately, this adaptable and scalable method guarantees that organizations, regardless of their size or sector, can efficiently streamline operations and better address patient requirements while reducing delays and enhancing patient results.

## 2. Methods

### 2.1 Systematic literature review process

**Figure 2.**
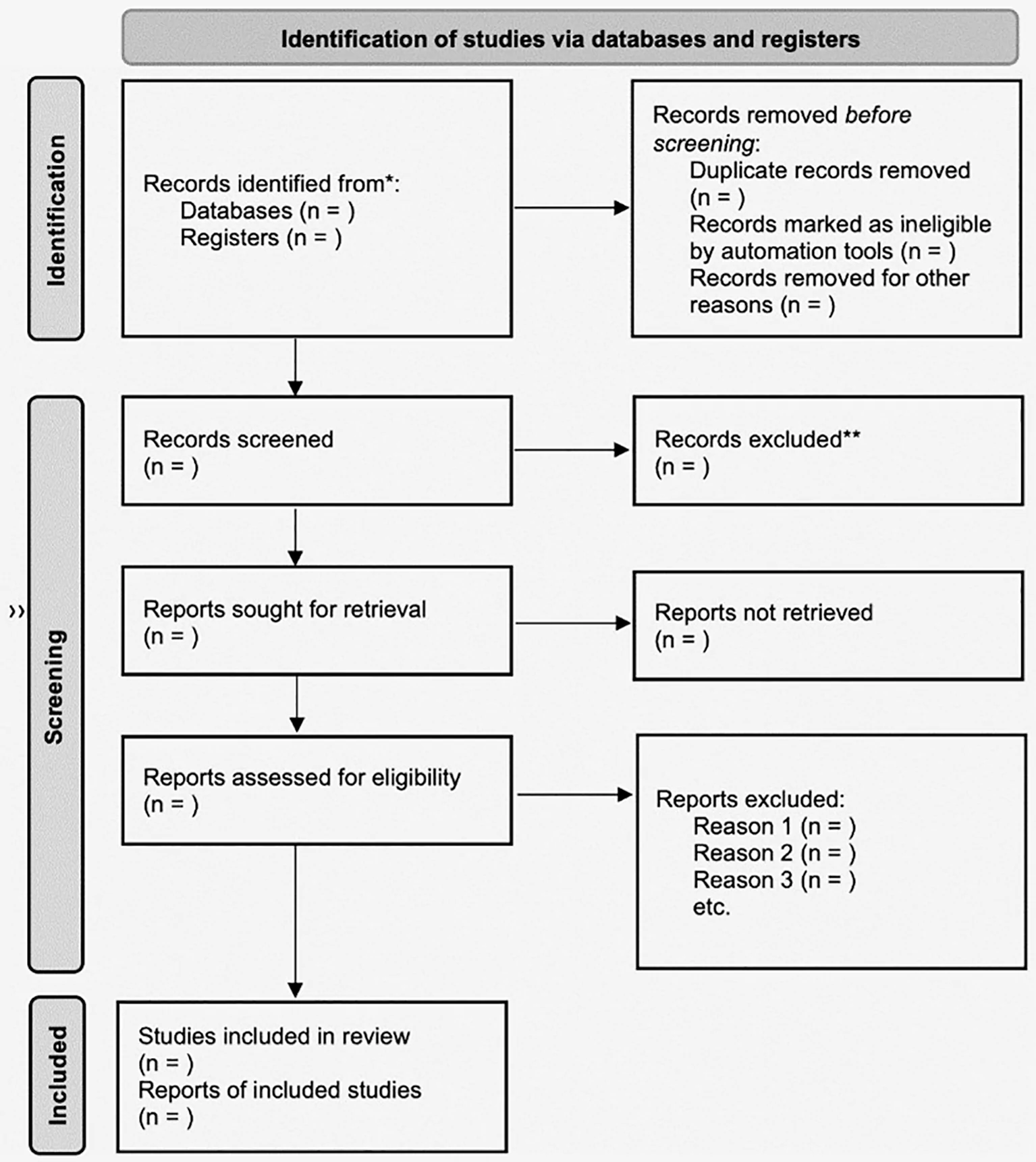
PRISMA guideline flowchart.

This research utilized a systematic literature review (SLR) in accordance with the Preferred Reporting Items for Systematic Reviews and Meta-Analyses (PRISMA) framework ^13^ to guarantee an organized and reproducible review procedure. The PRISMA framework has gained extensive acceptance in health related research, encompassing fields like telerehabilitation, confirming its relevance to studies on healthcare technology. In this study, the PRISMA flowchart ^14^ was used, as shown in Figure 2. To improve the thoroughness of the review, various data sources have been examined. Alongside searches in formal academic databases, Google searches were conducted to include pertinent literature that could not be indexed in prominent journal repositories. This approach granted access to various sources, including books, book chapters, institutional websites, and gray literature, facilitating a more comprehensive review.

### 2.2 Database selection and search strategy

This systematic review focused on seven major academic databases specializing in healthcare, technology, and management. The title and abstract screening process was used to filter out irrelevant studies, with a focus on research exploring real-time monitoring and tracking systems specifically designed to reduce patient Turnaround Time (TAT) in hospital settings. Studies that did not align with the core criteria were excluded. Table 1 lists the databases included.

**Table 1.** Database selection.

Database	Descriptions
Emerald Insight	Medical and healthcare related studies
SpringerLink	Applied sciences and healthcare
DOAJ	Multidisciplinary and healthcare
ScienceDirect	Multidisciplinary journals
PubMed	Multidisciplinary and healthcare journals
IEEE Xplore	Technological and IoT based research
Google Scholar	Ensure inclusion of additional relevant studies

### 2.3 Inclusion and exclusion criteria

The inclusion criteria were designed to capture studies that:

• Real-time patient location tracking technologies are implemented, including Bluetooth low-energy (BLE) systems.
• Demonstrate measurable improvements in TAT and hospital operational efficiency.
• Focus on public healthcare environments, particularly in Malaysia or other similar settings.

Exclusion criteria eliminated:

• Studies unrelated to IoT based solutions or those focused on non healthcare applications.
• Research outside the timeline of 2017 to 2024 to maintain relevance and capture recent technological advancements.

### 2.4 Timeframe and scope

This review examines publications spanning 2017 to 2024, a timeframe selected to capture the rapid advancements and growing adoption of IoT technologies within the healthcare sector. This period reflects the evolution and integration of innovative Bluetooth low-energy (BLE)-based systems, which have significantly influenced hospital efficiency and patient care management. By focusing on this era of technological transformation, the review ensures the inclusion of cutting edge solutions and trends that align with Malaysia’s ongoing healthcare improvement initiatives. The primary objective of this systematic review is to synthesize existing research, providing a comprehensive analysis of the impact of IoT enabled systems on hospital operations. It highlights key findings, uncovers recurring themes, and identifies critical knowledge gaps that present opportunities for innovation. This study aimed to offer actionable insights into how BLE and IoT technologies can streamline workflows, enhance resource management, and optimize patient turnaround times in public hospitals.

Ultimately, this review contributes to the development of advanced hospital management systems that not only reduce operational inefficiencies, but also elevate the overall quality of healthcare delivery. By addressing existing challenges and paving the way for further innovation, the findings can guide policymakers, administrators, and technologists in building a connected, efficient, and patient-centered healthcare ecosystem.

## 3.0 Analysis and results

### 3.1 Systematic literature review for absence of real time patient location monitoring and tracking solution in medical facilities

The SLR seeks to collect, examine, and integrate current research to offer comprehensive insight into the fundamental elements necessary for real-time patient location monitoring and tracking solutions. This analysis aimed to identify the essential factors that enhance patient turnaround times in healthcare settings. A Systematic Literature Review (SLR) offers a comprehensive analysis of current studies to identify best practices, technological advancements, and the main issues recognized by specialists in the domain. This examination provides an essential understanding of how real-time tracking systems can optimize patient movement, improve operational productivity, and elevate the overall service provision in healthcare settings. In addition to highlighting their strengths, the review reveals shortcomings in existing solutions, providing insights into the key features necessary to enhance or create these systems. ^15^ This guarantees that the solutions correspond to the evolving requirements of public hospitals and medical institutions, tackling both current and upcoming issues.

The findings from the SLR contribute to a more informed understanding of the technical, organizational, and patient-centered factors that impact the effectiveness of real-time patient location monitoring and tracking solutions. This knowledge serves as the foundation for designing comprehensive solutions tailored to meet the evolving needs of healthcare operations. Integrating these insights with other components of the research will ensure a well-rounded approach, helping to achieve the study’s objectives and driving meaningful improvements in hospital performance and patient outcomes. The absence of a comprehensive framework may suggest that smaller, more specific indexes, such as those for real-time patient location monitoring and tracking solutions, are also unlikely to exist. Even if such solutions or frameworks exist, thorough research is still necessary to determine whether this segment has been adequately explored in the existing literature.

These two indicators (the Smart Healthcare definition and the Smart Healthcare Index) play a crucial role in assessing whether the foundational groundwork has been laid for real-time patient location monitoring and tracking solutions to emerge in academic and industry discussions. If these indicators are well-defined, they can serve as signposts, indicating that the topic is ready to be further explored. Conversely, the absence of these indicators highlights the need for further research and development before such solutions can gain wider acceptance or visibility. Following this, a direct investigation into the existence of a real-time patient location monitoring and tracking index will be conducted, even if the initial two indicators are not fully met. This ensures that no existing frameworks or research contributions are overlooked. This section provides an in-depth review of the literature on both the definition and index, as well as an extensive analysis of existing studies related to real-time patient location monitoring and tracking solutions. This combined approach offers a clear understanding of the current state of knowledge and identifies the gaps that need to be addressed for future development.

The two key indicators Smart Healthcare’s comprehensive definition and Smart Healthcare Index were absent in the literature, indicating that an index for real-time patient location monitoring and tracking solutions is also unlikely to exist. This conclusion was confirmed through searches of major academic journals, as summarized in Table 2. A Systematic Literature Review (SLR) was conducted to address objective (2) using two specific keywords: (1) real-time patient location monitoring and the tracking solution index. The keywords were chosen to ensure that potential research was captured, even if the solutions existed outside a “smart” environment framework. The search covered both smart and non-smart settings to provide a more comprehensive view.

**Table 2.** Result search of patient real time location monitoring and tracking solution in healthcare facilities in major journal databases.

No.	Paper/Journal database and search engine	Number of results	Number of related results (s)
Keywords: Patient real time location monitoring and tracking solution			
1.	Emerald Insight	155	0
2.	Springer	65	0
3.	DOAJ	10	0
4.	Science Direct	281	0
5.	PubMed	91	0
6.	IEEE Explore	61	0
7.	Google Scholar	35	0

### 3.2 Choice of database

In a Systematic Literature Review (SLR), a comprehensive search across multiple databases is essential to ensure the quality and depth of the review. ^16^ Accessing major journal databases is crucial, as they provide peer-reviewed, high-quality studies relevant to specialized fields, such as healthcare, engineering, and information systems. For this study, searches were performed in the following key databases to gather relevant literature for developing a real-time patient location monitoring and tracking solution in healthcare facilities: Directory of Open Access Journals (DOAJ), ScienceDirect, SpringerLink, PubMed, IEEE Xplore, Emerald Insight, and Google Scholar. These databases ensure a broad coverage of both theoretical and practical studies and offer valuable insights needed to build an effective solution.

These databases were strategically selected for extensive coverage across disciplines relevant to this study. Each platform offers unique insights that are essential for developing real-time patient location monitoring and tracking solutions. DOAJ and PubMed focus on healthcare systems and patient care, providing valuable perspectives on clinical practices and patient-centered approaches. ScienceDirect and SpringerLink cover technological innovations and engineering solutions that are crucial for understanding the technical infrastructure behind real-time tracking systems. IEEE Xplore specializes in information and communication technology (ICT) applications, offering research on sensor networks, IoT devices, and real-time data transmission, which are all key components of patient monitoring solutions. Emerald Insight provides insights into healthcare management and operational efficiency, helping to explore how real-time tracking can optimize patient flow and reduce turnaround times. This diverse selection ensures a comprehensive review integrating technical, clinical, and management perspectives, which is essential for building an effective real-time patient tracking system.

By searching across this broad spectrum of databases, we ensured a well-rounded collection of resources that encompass both the technical and operational aspects of real-time patient location monitoring and tracking solutions. This comprehensive approach is expected to yield a significant number of high-quality studies that not only reflect current trends and challenges in healthcare technology, but also contribute valuable insights into the development and optimization of such systems in medical facilities. Additionally, this thorough search allowed us to identify potential research gaps, ensuring that the review offers both a solid foundation and innovative directions for future work in this field.

### 3.3 Search terms and eligibility criteria

For this study on real-time patient location monitoring and tracking solutions in healthcare facilities, two primary search terms were identified: “patient real-time location monitoring” and “tracking solutions in medical facilities.” These keywords were carefully chosen to align with the study’s core objectives, emphasizing the capabilities required for healthcare professionals to track and monitor patients in real-time within hospital settings. The search was conducted across multiple databases, and the initial results were screened by reviewing titles and abstracts to determine their relevance. Studies that did not meet predefined criteria were excluded to streamline the process. Only studies that met the eligibility requirements, specifically focusing on patient location monitoring systems, their implementation, and relevant tracking technologies, were included for further analysis. This approach ensured that the review remained focused and aligned with the objectives of the study.

In the subsequent stages, the selected studies were examined more closely, focusing on identifying key elements related to the functioning conditions, attributes, and operational environments of real-time patient location monitoring systems. Studies that demonstrated a strong connection to research focus, especially in terms of technological integration, patient care impact, and hospital operational efficiency, were subjected to deeper scrutiny. This in-depth review sought to uncover valuable insights into how these tracking solutions function in practical healthcare settings, the challenges they address, and the potential benefits they offer to improve patient flow, reduce turnaround times, and enhance the overall healthcare service quality. By applying this structured approach to search terms and literature selection, the study was able to avoid information overload while ensuring that only the most relevant and impactful studies were included in the review. This process not only narrowed the focus, but also enabled a thorough exploration of the key components that make real-time patient location monitoring and tracking solutions essential in medical facilities.

## 4. Results and discussion using meta analysis on SLR

As shown in Table 2, none of the reviewed databases contained a specific index paper related to real-time patient location monitoring and tracking solutions. Although several prominent journal databases returned multiple search results, none of the retrieved documents were directly relevant or applicable to the focus of this study. This lack of pertinent literature reveals a significant research gap, indicating that no established real-time patient location monitoring and tracking solution index currently exists within the academic or research communities. This conclusion was further supported by two critical findings.

• Extensive searches across multiple databases did not reveal any comprehensive or systematically developed framework that could guide researchers or practitioners in this field.
• Despite using diverse search terms and methodologies, the results failed to produce a cohesive body of work outlining the fundamental components of such solutions.

These findings emphasize the need for future research to fill this gap and develop a robust framework for real-time patient-tracking solutions in healthcare environments. These indicators underscore the current limitations in the available research, emphasizing the need for a dedicated body of literature that addresses real-time patient location monitoring and tracking solutions holistically. The creation of a formalized index or framework would be instrumental in guiding future research and development efforts in this emerging field, as well as aid healthcare facilities in implementing efficient patient-tracking technologies to improve operational efficiency and patient outcomes.

**Figure 3.**
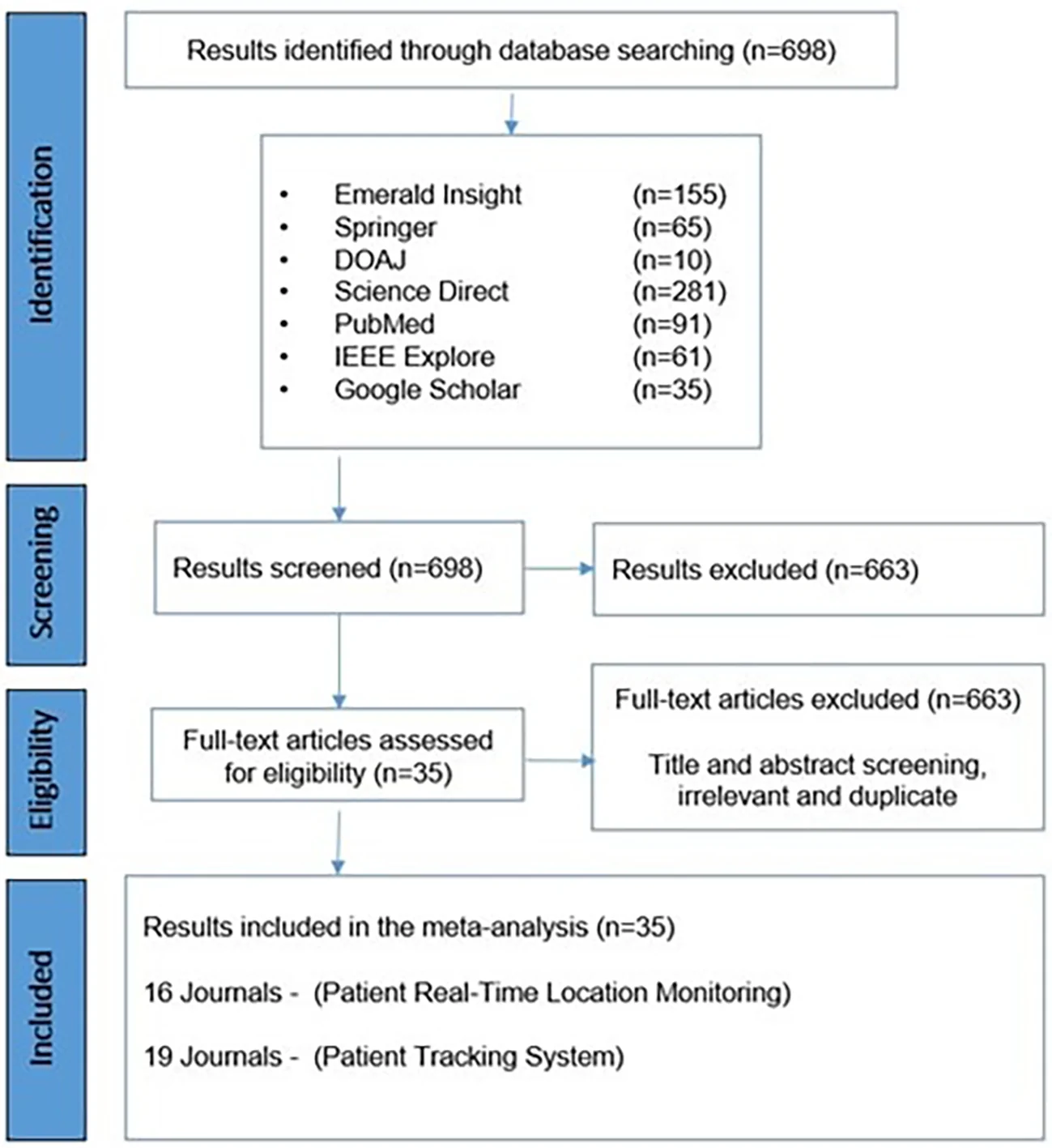
Systematic literature review results for real time patient location monitoring and tracking solution.

Following the PRISMA guidelines, ^14^ the Systematic Literature Review (SLR) identified a total of n=698 studies from the selected databases, as illustrated in Figure 3. This large volume of initial results highlights the growing interest and advancements in real-time patient location monitoring and tracking solutions, as well as the recognition of their importance in enhancing patient care and operational efficiency in healthcare settings. However, after the first screening step, which involved removing duplicates, reviewing titles and abstracts, and applying eligibility criteria, the number of relevant studies was narrowed to n=35. This refinement process ensured that only the most relevant, high-quality studies were included in the final dataset. A key consideration during this phase was the availability of full-text articles, with preference given to open-access papers to facilitate accessibility without subscription barriers. After applying these additional filters, 35 studies were selected for further analysis to form the core dataset for this review.

This final set of 35 articles provides a robust and comprehensive foundation for analyzing the state of real-time patient location monitoring and tracking solutions in healthcare facilities. Although reduced from the initial dataset, this number is still significant and allows for detailed exploration of the key themes, trends, and challenges within the field. Moreover, the chosen studies cover a broad spectrum of perspectives, ranging from technological innovations to the operational and clinical impacts of patient tracking systems. The literature reviewed highlights a recurring issue in medical facilities patients being subjected to extended wait times and occasional lapses in treatment. These challenges are often attributed to the overwhelming volume of daily cases, which creates a substantial workload for the healthcare staff. Managing patient logistics, administering treatment, prescribing medications, and handling other tasks requires significant coordination, especially in high-traffic hospital environments. As the volume of patients increases, the demand for healthcare workers also increases, putting additional strain on hospital resources.

This group of 35 articles offers a strong and thorough basis for examining the status of real-time patient location monitoring and tracking solutions in healthcare settings. Although it decreased from the original dataset, this figure remains important and enables in-depth examination of the primary themes, trends, and challenges in the discipline. Additionally, the selected studies encompassed a wide variety of viewpoints, from technological advancements to the operational and clinical effects of patient monitoring systems. The reviewed literature underscores a common problem in healthcare facilities, with patients facing prolonged wait periods and sporadic treatment gaps. These difficulties are frequently linked to an excessive number of daily cases, resulting in a significant burden on the healthcare personnel. Coordinating patient logistics, delivering treatment, prescribing drugs, and managing various tasks demands considerable organization, particularly in busy hospital settings. With the growing number of patients, the need for healthcare staff escalates, creating more pressure on hospital resources.

This meta-analysis examines the selection and screening methodology utilized in a Systematic Literature Review (SLR) focused on research pertaining to patient real-time location monitoring and tracking solutions. Following the PRISMA guidelines, ^14^ the methodology included a comprehensive search, screening, and refinement process to identify the most relevant studies for the final review. The findings revealed both the volume of research in this area and the methodology employed to narrow the selection. A total of 698 studies were initially identified from various databases, indicating a substantial amount of research in this field. This significant number reflects growing interest in technology-driven healthcare solutions, particularly concerning patient location tracking within medical facilities. This surge in research can be attributed to several factors such as enhanced operational efficiency in hospitals, improved patient care through better monitoring, and the development of advanced technological solutions.

The emphasis on patient location tracking and monitoring systems highlights their potential to optimize hospital workflows and enhance safety measures, minimizing the risks of lost patients and inefficient resource allocation. The first screening step involved eliminating duplicates, reviewing titles and abstracts, and applying eligibility criteria, resulting in a reduction of relevant studies from 698 to n=35, representing a 95% reduction in the initial dataset. This substantial decrease underscores the significance of rigorous screening criteria to ensure that only high-quality peer-reviewed studies are included. After applying all filters, 35 studies were selected for detailed review. This curated collection not only focuses on the specific technologies and systems utilized for patient tracking but also reflects broader trends in healthcare digitalization. The selected studies likely encompass rich data on technology implementations (e.g., RFID, Bluetooth, IoT systems), healthcare outcomes, such as reduced turnaround times and improved patient flow management, and cost-benefit analyses related to the deployment of real-time monitoring solutions.

The systematic literature screening process detailed in this review underscores the critical role of structured methodologies such as PRISMA in refining and analyzing extensive datasets. Beginning with an initial pool of 698 studies, the application of stringent screening criteria systematically narrowed the scope to 35 highly relevant studies. This significant reduction highlights the necessity to adopt robust, transparent, and replicable methodologies to ensure that only the most pertinent and high-quality research is included in the final analysis. This rigorous approach enables researchers to draw meaningful and reliable conclusions regarding the implementation and effectiveness of real-time patient location-monitoring systems in healthcare. By focusing on studies that meet defined quality and relevance thresholds, this review ensures that the insights derived are both credible and actionable, paving the way for data-driven decision-making in hospital management and healthcare policy.

Moreover, the findings reinforce the growing importance of integrating advanced tracking technologies such as IoT and BLE in modern healthcare systems. These technologies not only streamline patient workflows, but also enhance operational efficiency, resource management, and patient satisfaction. The systematic methodology also highlights gaps in current research, providing a roadmap for future studies to address unexplored areas such as long-term impacts, scalability, and integration challenges. By demonstrating the value of a meticulous screening process, this review not only validates the effectiveness of real-time tracking systems in improving patient outcomes but also advocates for their widespread adoption as a cornerstone of connected and efficient healthcare ecosystems.

## 5. Conclusion

In conclusion, the discussions in this section highlight a critical gap: there is currently no universally accepted or comprehensive definition of Smart Healthcare, which is essential for establishing foundational metrics, such as real-time patient location monitoring and tracking solution index. Despite extensive research on Smart Healthcare applications, the lack of a standardized definition has hampered the creation of a clear framework for advanced healthcare solutions, particularly regarding real-time patient monitoring and tracking. This deficiency likely arises from the common assumption that Smart Healthcare is solely defined by the integration of smart technologies, neglecting their scope, boundaries, and contextual relevance. The absence of a unified definition has far-reaching implications for future research. This not only indicates the lack of a Smart Healthcare Index but also suggests that no standardized real-time patient location monitoring and tracking solution index currently exists. Without such frameworks, the fundamental principles, components, and benchmarks that define “smartness” in healthcare remain ambiguous, complicating stakeholders’ efforts to assess the effectiveness and maturity of healthcare innovations in this domain.

Moreover, our investigation revealed a second critical indicator: the Smart Healthcare Index was missing. This is evident from the scarcity of related academic literature, with only one relevant paper identified from a conference highlighting the limited scholarly focus on this area. The notable absence of both a clear definition and structured index strongly affirms the non-existence of a real-time patient location monitoring and tracking solution index. This finding substantiates the Problem Statement, emphasizing the urgent need for further research, development, and standardization in this field. To bridge these gaps, future research should prioritize the development of a comprehensive and standardized definition of Smart Healthcare considering its various dimensions, including technological, clinical, and operational aspects. Establishing this framework will enhance the understanding and lay the groundwork for creating indices that can effectively guide research, implementation, and policymaking in this critical area. Addressing these challenges is vital for advancing real-time patient-tracking technologies, which have the potential to significantly improve patient outcomes, streamline operations, and elevate overall healthcare quality.

The growing influx of patients in hospitals poses significant challenges for healthcare facilities across the country. ^17^ With an increased focus on patient turnaround time (PTAT), the impact of prolonged patient stays, such as rising costs and lower patient satisfaction, becomes more evident, particularly in light of increasing patient volumes. Hospitals are actively exploring effective solutions to address these concerns. This study investigated a hospital-wide initiative aimed at enhancing PTAT. Various research methods, including a literature review, have been employed to assess turnaround times. The key issues identified include tracking and communication between departments, interactions between staff and patients, and the movement of patients throughout the hospital, which can result in missed treatments. These findings underscore critical areas that require attention to boost operational efficiency and improve patient care.

Assessing medical workflows via turnaround times continues to be difficult because of the different definitions within the same healthcare environment. Reaching an average patient turnaround time (PTAT) target of 90 min or less per visit can provide substantial advantages for both hospital administrators and patients. Nonetheless, Malaysia’s Ministry of Health (MOH) observed in 2017 that public hospitals in Malaysia still encounter difficulties in providing quality services, especially concerning patient turnaround times. This situation is especially troubling, because numerous public healthcare institutions find it difficult to satisfy these PTAT criteria, resulting in a drop in care quality. As stated in a report by Malay Mail in 2021, many patients frequently depart without treatment after waiting for more than 90 minutes. ^18^ This scenario presents extra difficulties for healthcare providers, as doctors might struggle to ascertain whether a patient remains in the facility or has returned home. These inefficiencies impact patient results and heighten public concern regarding the quality of healthcare services in Malaysia.

Intended as a readily available and accessible solution for standard cellphone networks, this approach faces challenges owing to magnetic field fluctuations, which render movement data collected via cellphones unreliable and inaccurate. Another article ^19^ discussed a solution centered on specialized hardware sensors, but did not specify the technology employed. Similarly, an article ^20^ proposed wireless and mobile networks as theoretically suitable solutions, but offered no concrete implementations. The meta-analysis revealed that no existing studies have explored improving turnaround times through real-time patient location tracking using BLE-based IoT technology. These innovations aim to significantly reduce patient turnaround time (PTAT) and enhance customer satisfaction. ^21^ The absence of practical solutions underscores a critical gap in the current body of research, and highlights the need for further investigation in this field.

## Ethics and consent

Ethical approval and consent were not required.

## Data Availability

Figshare: Experimentation Data: https://doi.org/10.6084/m9.figshare.28473533.v1 ^22^ Figshare: Figure: https://doi.org/10.6084/m9.figshare.28824263.v1 ^23^ Data are available under the terms of the Creative Commons Zero “No rights reserved” data waiver (CC0 1.0 Public domain dedication). Figshare: PRISMA Check List for “An IoT BLE Based System Literature Review of Real Time Location Monitoring and Tracking to Shorten Patient Wait Times in Malaysian Public Hospitals”: https://doi.org/10.6084/m9.figshare.28724372.v1 ^24^ Data are available under the terms of the Creative Commons Zero “No rights reserved” data waiver (CC0 1.0 Public domain dedication).
