# Supplementary material for: An IoT BLE based system literature review of real time location monitoring and tracking to shorten patient wait times in Malaysian public hospitals
Source: F1000Res. 2025 Jun 9;14:568. [Version 1] doi: 10.12688/f1000research.160992.1 (PMC12449682; doi:10.12688/f1000research.160992.1)
Supplement: Peer review history

# Reviewer report: Reviewer response for version 1

**DOI:** 10.5256/f1000research.176964.r402589

**Published:** 2026-08-14

**Authors:** Omid Panahi

**Affiliations:** 1 Yeditepe University, Istanbul, Turkey

This manuscript addresses an important healthcare management issue: reducing patient turnaround time (PTAT) in Malaysian public hospitals through the potential use of IoT- and Bluetooth Low Energy (BLE)-based real-time patient tracking systems. The topic is timely and relevant, particularly given increasing patient volumes and the growing adoption of digital health technologies. The authors conducted a systematic literature review (SLR) using the PRISMA framework and identified challenges related to patient flow, tracking, and operational efficiency in healthcare settings.

 While the manuscript highlights a relevant problem and presents a potentially valuable technological solution, several methodological and reporting limitations reduce the scientific rigor of the review and weaken the strength of the conclusions.

 Major Issues Requiring Revision

**1. Incomplete reporting of the systematic review process**

 Although PRISMA is cited and a flow diagram is provided, the search strategy is insufficiently described. The manuscript does not provide complete search strings, database-specific queries, screening procedures, reviewer roles, or methods used to resolve disagreements during study selection. These details are necessary for reproducibility and should be added.

**2. Inconsistency between the number of included studies and cited literature**

 The review reports that 35 studies were included after screening; however, the reference list contains substantially fewer studies directly related to the final dataset. The relationship between the 35 included studies and the cited references is unclear. A table summarizing all included studies, their characteristics, methodologies, and key findings should be provided.

**3. Lack of a true systematic synthesis**

 The manuscript mainly provides a narrative discussion rather than a structured synthesis of the included studies. There is limited comparative analysis of technologies, implementation outcomes, and evidence quality. The authors should include summary tables and thematic analyses describing:

- Technology used (BLE, RFID, Wi-Fi, UWB, etc.).
- Healthcare setting.
- Sample size and study design.
- Reported impact on waiting time, PTAT, workflow efficiency, or patient outcomes.
- Limitations of each study.

**4. Conclusions exceed the available evidence**

 The manuscript proposes that BLE-based systems could reduce waiting times by 35% and achieve PTAT below one hour. However, this specific performance claim is not adequately supported by the presented review findings. The conclusions should be revised to clearly distinguish between evidence derived from the literature and the authors' proposed future solution.

**5. Absence of quality assessment**

 No formal quality appraisal or risk-of-bias assessment of the included studies is reported. Established tools should be used where appropriate to assess the quality of the evidence and strengthen the validity of the review findings.

 Minor Issues

- The title could be more precise and explicitly indicate that this is a systematic literature review.
- Additional recent literature from 2024–2025 should be incorporated.
- Greater comparison between BLE and alternative tracking technologies (e.g., RFID, UWB, Wi-Fi localization) would strengthen the justification for selecting BLE.
- Several sections contain repetitive discussions and could be streamlined for clarity.
- Figures and tables could be expanded to better illustrate workflow improvements and expected operational benefits.

Overall Assessment

 The manuscript addresses a meaningful healthcare challenge and identifies an important gap in patient-tracking solutions within public hospitals. However, significant improvements in methodological transparency, evidence synthesis, study quality assessment, and interpretation of findings are required before the conclusions can be considered fully supported by the review. Addressing the issues outlined above would substantially improve the scientific rigor and practical value of the article.

**Recommendation: Major Revision**.

Are the rationale for, and objectives of, the Systematic Review clearly stated?

Partly

Is the statistical analysis and its interpretation appropriate?

Not applicable

If this is a Living Systematic Review, is the ‘living’ method appropriate and is the search schedule clearly defined and justified? (‘Living Systematic Review’ or a variation of this term should be included in the title.)

Not applicable

Are sufficient details of the methods and analysis provided to allow replication by others?

Partly

Are the conclusions drawn adequately supported by the results presented in the review?

Partly

Reviewer Expertise:

Dentistry, Oral Health Research, Healthcare Informatics, Artificial Intelligence in Healthcare, Digital Health Technologies, Evidence-Based Medicine, Systematic Reviews and Meta-Analysis, and Health Services Research

I confirm that I have read this submission and believe that I have an appropriate level of expertise to confirm that it is of an acceptable scientific standard, however I have significant reservations, as outlined above.

# Reviewer report: Reviewer response for version 1

**DOI:** 10.5256/f1000research.176964.r399362

**Published:** 2025-09-19

**Authors:** Noor Zaman Jhanjhi

**Affiliations:** 1 Taylor's University, Subang Jaya, Selangor, Malaysia

- **Novelty and Significance**
  The manuscript addresses an important question. The approach is original, and the findings offer new insights that advance current understanding. It fills a gap in the literature, and has potential implications for future research.
- **Methodological Rigor**
  The experimental / analytic methods are well thought out and executed. Controls (or comparative models) are appropriate; the data collection is thorough and of high quality. Statistical analyses are sound and transparently reported.
- **Clarity and Organization**
  The overall structure of the paper is logical and user-friendly. The introduction sets up the problem nicely, the methods are described in enough detail to allow reproducibility, and the results are presented clearly. The figures/tables are high quality and support the text well.
- **Balanced Interpretation**
  The discussion interprets the results carefully without overstating conclusions. The authors acknowledge limitations where appropriate and suggest future directions. This adds credibility.
- **Relevance and Broader Impacts**
  The findings have relevance beyond the immediate study system. The authors make a compelling case for broader implications, potential applications, or how this work could stimulate further lines of inquiry.

Are the rationale for, and objectives of, the Systematic Review clearly stated?

Yes

Is the statistical analysis and its interpretation appropriate?

Not applicable

If this is a Living Systematic Review, is the ‘living’ method appropriate and is the search schedule clearly defined and justified? (‘Living Systematic Review’ or a variation of this term should be included in the title.)

Yes

Are sufficient details of the methods and analysis provided to allow replication by others?

Yes

Are the conclusions drawn adequately supported by the results presented in the review?

Yes

Reviewer Expertise:

My expertise lies in cybersecurity, IoT security, wireless networks, and data protection, with strong integration of artificial intelligence and machine learning methods. His recent work applies these to areas such as malware detection, intrusion detection, healthcare AI, and social media analytics, reflecting both technical depth and interdisciplinary reach.

I confirm that I have read this submission and believe that I have an appropriate level of expertise to confirm that it is of an acceptable scientific standard.

# Reviewer report: Reviewer response for version 1

**DOI:** 10.5256/f1000research.176964.r405327

**Published:** 2025-09-04

**Authors:** Sangeetha Rangasamy

**Affiliations:** 1 Christ University Bengaluru, Bengaluru, India

The authors' initiative in addressing an important societal problem with a possible IoT-based solution through their research is very much appreciated. 

 However, addressing the following minor issues will add more value to the scholarly work.

 1. The title of the manuscript needs a revision - BLE to be expanded, SLR can be used for Systematic Literature Review since BLE may not be known to all the readers. 

 2. Review of literature needs improvement. A few more literature pertaining to 2025 can be added.

 3. PRISMA model highlights that there are 35 articles finalized for meta-analysis, but the references provided are 24. Does it mean that not all the articles are included to prepare the content of the article? 

 4. A few more tables can be presented to understand citations and their relevance. 

 5. Figure 1 needs improvement in terms of the expected outcome of the IoT implementation. Checkpoints and reduction in time compared with the present state adds more understanding.

Are the rationale for, and objectives of, the Systematic Review clearly stated?

Yes

Is the statistical analysis and its interpretation appropriate?

Yes

If this is a Living Systematic Review, is the ‘living’ method appropriate and is the search schedule clearly defined and justified? (‘Living Systematic Review’ or a variation of this term should be included in the title.)

Yes

Are sufficient details of the methods and analysis provided to allow replication by others?

Yes

Are the conclusions drawn adequately supported by the results presented in the review?

Yes

Reviewer Expertise:

IoT/BCT in Healthcare, Banking, Education. Macro economic and banking, Sustainable and climate finane.

I confirm that I have read this submission and believe that I have an appropriate level of expertise to confirm that it is of an acceptable scientific standard, however I have significant reservations, as outlined above.

# Response

**Published:** 2025-09-12

**Authors:** NZ Jhanjhi

**Affiliations:** Computer Science, Taylor's University, Subang Jaya, Selangor, Malaysia

The article is now in better shape to be accepted for indexing

# Reviewer report: Reviewer response for version 1

**DOI:** 10.5256/f1000research.176964.r390787

**Published:** 2025-07-28

**Authors:** Souvil Pal

**Affiliations:** 1 Sister Niveda University, Rajarhat, India; 2 Department of Computer Science and Engineering, Sister Niveda University, Newtown, West Bengal, India

A. Malaysia’s public hospitals are facing some serious challenges right now—long waiting times, staff shortages, and systems that just aren’t keeping up. This study explores how Bluetooth Low Energy (BLE) technology could help ease that pressure and streamline hospital operations. It’s not some far-off concept—it’s a practical solution that could address real problems happening today.

 B. The research team took a methodical approach, following the PRISMA framework to guide their review. They pulled data from top-tier databases like IEEE and PubMed, focusing on studies published between 2017 and 2024. This helped them zero in on what’s really causing the bottlenecks—why patients are waiting so long in the first place.

 C. One major finding stood out: hospitals don’t have a consistent, tech-based way to track patients throughout their care journey. That’s a big gap. Instead of stopping there, the team went deeper. They analyzed 35 high-quality studies, drawing out key themes, patterns, and missed opportunities. It’s thoughtful, well-organized work with real-world value.

 D. The paper breaks down what a real-time tracking system would look like, using BLE tags, sensors, backend software, and staff dashboards. Even if you’re not tech-savvy, the layout is easy to follow. The diagrams showing patient flow through the hospital really help bring the concept to life.

 E. A BLE wristband system that could reduce patient turnaround time by up to 35%. That means shorter waits, fewer missed appointments, and smoother operations overall. It’s a smart, realistic fix that could make a meaningful difference for both hospital staff and the patients they serve.

Are the rationale for, and objectives of, the Systematic Review clearly stated?

Yes

Is the statistical analysis and its interpretation appropriate?

Yes

If this is a Living Systematic Review, is the ‘living’ method appropriate and is the search schedule clearly defined and justified? (‘Living Systematic Review’ or a variation of this term should be included in the title.)

Yes

Are sufficient details of the methods and analysis provided to allow replication by others?

Yes

Are the conclusions drawn adequately supported by the results presented in the review?

Partly

Reviewer Expertise:

Data Analytics, Cloud Computing, IoT, Sensors

I confirm that I have read this submission and believe that I have an appropriate level of expertise to confirm that it is of an acceptable scientific standard.
